# Analysis of a Pediatric Dental School Patient Population Revealed Increasing Trends of Limited English Proficiency (LEP) Patients: Implications for Pediatric Dental Public Health and Access to Care

**DOI:** 10.3390/pediatric14020035

**Published:** 2022-06-02

**Authors:** Jasnena Mavi, Karl Kingsley

**Affiliations:** 1 Department of Advanced Education in Pediatric Dentistry, School of Dental Medicine, University of Nevada-Las Vegas, 1700 W. Charleston Boulevard, Las Vegas, NV 89106, USA; mavij1@unlv.nevada.edu; 2 Department of Biomedical Sciences, School of Dental Medicine, University of Nevada-Las Vegas, 1001 Shadow Lane, Las Vegas, NV 89106, USA

**Keywords:** Limited English Proficiency (LEP), pediatric, dental

## Abstract

Based upon the lack of current information regarding the pediatric patient population at UNLV-SDM, the overall goal of this project was to analyze the demographic characteristics of this population, indicators for socioeconomic status (SES), such as enrollment in Medicaid, and other barriers to healthcare access, such as non-English/non-Spanish languages spoken. Using an Institutional Review Board (IRB)-approved protocol, this analysis revealed the percentage of minority pediatric patients between 2010 and 2020 increased among African Americans, Asian Americans, and mixed or multiracial patients, while decreasing among Hispanics. Analysis of the Limited English Proficiency (LEP) patients and guardians found an overall increase in the number of non-English/non-Spanish languages spoken from *n* = 4 in 2010 to *n* = 21 in 2020 with no significant changes in Medicaid/CHIP enrollment identified between 2010 and 2020 (76.7%, 77.9%, *p* = 0.988). These data suggest the composition of the patient population has experienced significant shifts over time, with more patients of mixed racial backgrounds and increased numbers of Limited English Proficiency (non-English/non-Spanish foreign languages) spoken. These data may suggest there is an increased need for multilingual health materials, training, and translators for pediatric oral health within this population.

## 1. Introduction

Recent studies in pediatric dental public health have focused on prevention efforts and improved educational initiatives among pediatric healthcare providers to facilitate these efforts [1,2]. Much of the focus of these efforts has been dental caries, among the most prevalent and pervasive problems affecting children worldwide [3,4,5]. Many dental and oral health programs are now integrating dental public health prevention measures into the curriculum to maximize the potential for awareness and engagement in these efforts [6,7].

Among the most useful and effective tools to identify at-risk children has been the compilation and analysis of demographic and socioeconomic data, which have been successfully used to design and implement prevention strategies to reduce the burden of childhood caries in many lower socioeconomic status (SES) and underserved communities [8,9,10]. Accordingly, many of these programs rely on improving parental oral health literacy and engagement to improve frequency of care and prevention, as well as the development and maintenance of positive oral health behaviors among children [11,12]. However, recent evidence has demonstrated that factors other than low SES may also significantly contribute to these health disparities, including the role of language barriers and cultural differences among some racial and ethnic minorities [13,14].

Research from this institution has identified inequalities in caries risk and caries experience among pediatric patients in Nevada [15,16,17,18]. Most of these studies have confirmed the correlations between low SES with poor oral health outcomes among pediatric patients, although some efforts have been made to further understand the role of race, ethnicity and other factors, such as language barriers, in these populations [19,20]. However, most of these studies were conducted many years ago, and significant changes in the demographic, racial, ethnic and cultural makeup of the population have taken place [21,22,23,24].

For example, published epidemiologic data from Nevada between 2010 and 2020 have revealed that the percentage of White (non-Hispanic) patients has declined from 54.2% to 47.2%, while the proportion of other racial and ethnic minority groups has increased over this same time period [25,26]. More specifically, the percentage of Hispanics in Nevada increased from 26.6% to 29.5%, as did those who identified as Black (7.8% to 9.4%), Asian (7.2% to 8.5%), or multi-racial (2.8% to 3.7%) [27,28,29,30,31,32]. Recent studies have suggested that some of these minority populations in Nevada may include those who speak languages other than English or Spanish, which may further exacerbate health disparities and increase barriers to communication and healthcare access [33,34].

Based upon the lack of current information regarding the pediatric patient population at UNLV-SDM, the overall goal of this project was to determine the demographic characteristics of this dental school-based pediatric population (including sex, race/ethnicity and age), as well as indicators for socioeconomic status (SES), such as enrollment in public assistance or safety net services including Medicaid and other languages spoken. These data will be compared over time to the previous studies to identify any relevant changes or trends that may help towards designing and implementing appropriate prevention strategies for pediatric oral health.

## 2. Materials and Methods

### 2.1. Study Approval

This study was conducted according to the guidelines of the Declaration of Helsinki and was reviewed and approved as exempt by the University of Nevada, Las Vegas (UNLV) Institutional review board (IRB) under protocol [1619329-1] titled “Retrospective analysis of Oral Health Status of Dental Population” on 24 July 2020. This retrospective analysis of previously collected data regarding the UNLV School of Dental Medicine (SDM) pediatric patient population was deemed Exempt under Federal Regulation 45 CFR 46 and Informed Consent was waived pursuant to the Basic Health and Human Services (HHS) Policy for the Protection of Human Research Subjects (46.101) regarding IRB exemption for research that involves the study of existing data, documents or records that currently exist and are not prospectively collected and (1) Participants cannot be directly identified; and (2) Participants cannot be identified through identifiers linked to them.

### 2.2. Data Collection

De-identified demographic data, enrollment in public assistance programs (Medicaid/Children’s Health Insurance Program or CHIP) and languages spoken from all patients screened and treated at the UNLV-SDM pediatric patient clinic between 2010 and 2020 were obtained. Demographic data included sex or gender, racial and ethnic information (self-reported) to staff, and age of patient at time of initial visit. Demographic data are typically input during the initial screening or treatment visits by UNLV-SDM staff or employees, and can be updated or modified at any subsequent patient visit. For example, enrollment in public assistance programs including Medicaid and CHIP may occur after the initial patient screening or treatment visit and the most up-to-date information for each patient would be reflected in the retrospective data collection by the study authors, which occurred in 2020. Similarly, the need for translation services (non-English, non-Spanish speaking patients) could be updated or modified during any patient visit or encounter.

### 2.3. Data Analysis

Summary data for the UNLV SDM pediatric patient clinics over the period 2010–2020 were used to compile descriptive statistics (number and percentage) for the approved study demographic variables, such as sex, age, and race/ethnicity, as well as specific SES indicators (Medicaid, CHIP enrollment), and languages spoken (English, Spanish, or additional language as indicated). Analysis of any changes has been completed using Chi square analysis.

## 3. Results

The analysis of these retrospective data revealed a total of *n* = 24,849 pediatric patients between January 2010 and December 2020 (Table 1). From these patient data records, an analysis of sex was performed that determined complete records were found for 98.4% (*n* = 24,460/24,489) of all patients on file. These pediatric patients were approximately half female (52.2%) and half male (47.8%), which was not statistically significant, *p* = 0.6892. The percentage of patients with complete data for Race or Ethnicity was 77.6% (*n* = 19,281/24,849). Analysis of these data revealed the percentage of White (non-minority) patients was 19.3%, which is significantly lower than in the local population (47.4%), with the remainder of patients self-reporting as minority, *p* = 0.0001.

More specifically, the percentage of Hispanic and Black patients in the study sample was 52.4% and 12.2%, respectively, which was higher than their percentages in the local population (20.5% and 9.4%, respectively). However, the percentage of Asian/Pacific Islanders (3,8%) and Native Americans (0.1%) was lower than observed in the local population (9.2% and 0.8%, respectively). Finally, the percentage of patients reporting mixed race was 12.2%, which was slightly higher than the local population (10.2%). These data are significantly different from the distribution of minorities within the local population, *p* = 0.0001.

Further analysis revealed that complete data for age were available for 81.9% of all patients (*n* = 20,361/24,849). Analysis of these data revealed an average overall age of 9.04 +/−4.86 (STD) years, which ranged between 0 and 18 years old. The age distribution among this population ranged between a low of 8.2% (17–18 year olds, *n* = 1677/20,361) and a high of 14.4% (7–8 year olds, *n* = 2941/20,361).

**Figure 1:**
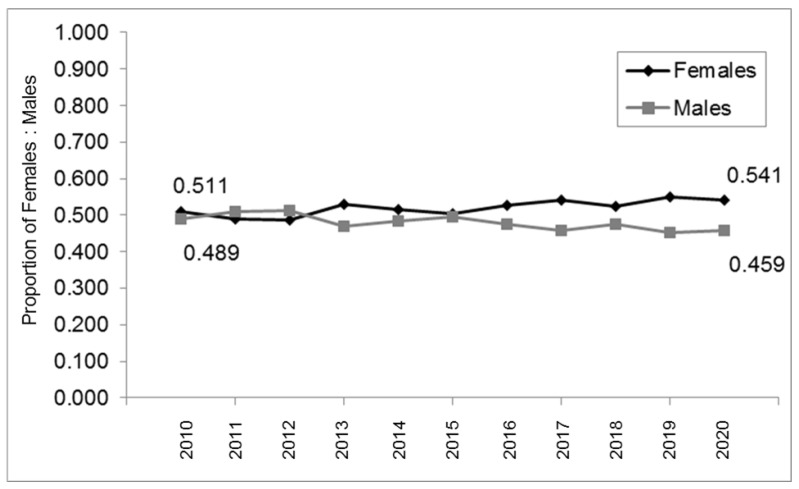
Proportion of males and females within the patient population over time. Plotting the proportion over time demonstrated relative stability among females (52%) and males (48%) between 2010 and 2020.

To evaluate if the proportion of females and males has changed over time, the data from Table 1 were graphed (Figure 1). These data demonstrate that the proportion of females and males appears to be relatively stable over time. More specifically, the proportion of females ranged between 48.8% and 54.9%—averaging 52.2% overall. The proportion of males ranged between 45.1% and 51.2%—averaging 47.9% overall.

**Figure 2:**
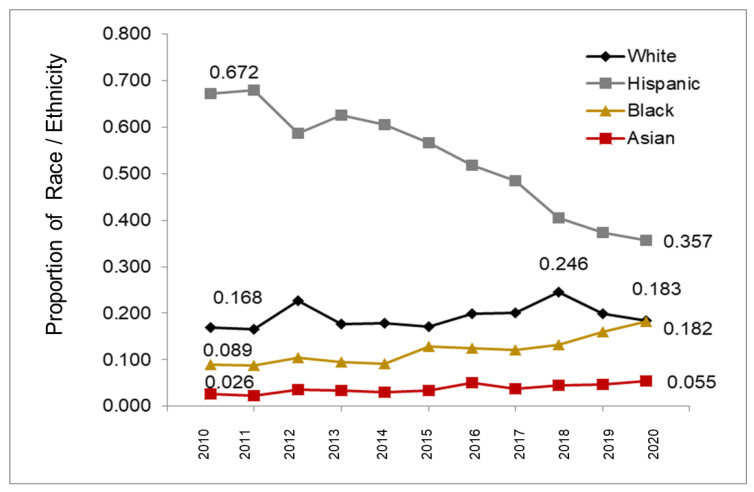
Change in proportion of racial and ethnic minorities within the patient population over time. The proportion of White/Caucasians remained fairly constant over time (16.8%, 2010 and 24.6%, 2018) although Hispanics decreased from 67.9% (2011) to 35.7% (2020). In contrast, the proportion of Black patients increased over time from 8.9% (2010) to 18.2% (2020), similar to the trend observed with Asians (2.6%, 2010 to 5.5%, 2020).

To determine any change over time in the proportion of racial and ethnic minorities, data from each year were plotted and graphed (Figure 2). These data demonstrated that although there was some variation in the proportion of White/Caucasians over time, the percentages remained fairly constant between 16.8% (2010) and 24.6% (2018), with an overall average of 19.3%. However, the proportion of Hispanics within this patient population appears to have changed from a high of 67.9% (2011) to a low of 35.7% (2020), a decrease of nearly 53%. In contrast, the proportion of Black patients within this population has increased over time from 8.9% (2010) to 18.2% (2020), an approximately 73% increase over this same time period. Finally, the proportion of Asians within this patient population has increased from 2.6% (2010) to 5.5% (2020)—a nearly two-fold increase.

**Figure 3:**
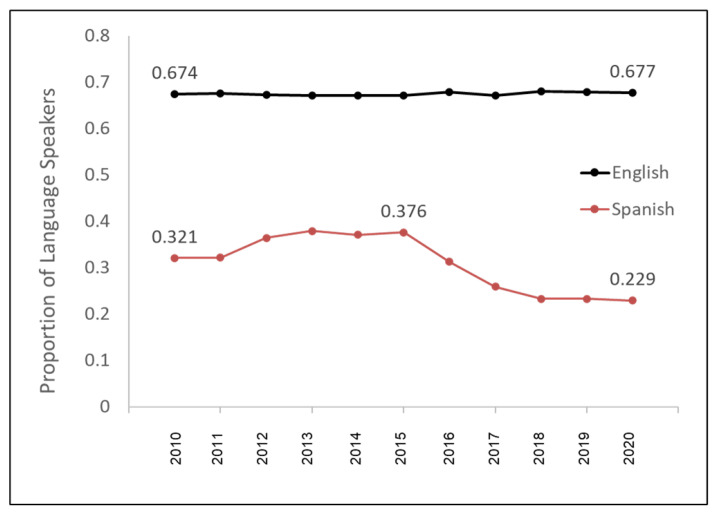
Change in the preferred language spoken over time. The majority of patients speak English as their preferred language, which remained relatively constant over time at approximately 67%. The percentage of patients who speak Spanish declined somewhat over time, decreasing from 32.1% (2010) to 22.9% (2020).

To evaluate if these changes in the relative proportion of racial and ethnic minorities were associated with any changes in the language spoken, these data were plotted and graphed (Figure 3). Analysis of these data revealed that the proportion of patients who speak English as their preferred language was relatively constant over time at 67.4% in 2010 to 67.7% in 2020. However, the relative proportion of patients who speak Spanish as their preferred language declined somewhat over time, decreasing from 32.1% in 2010 (with a high of 37.6% in 2016) to approximately 22.9% in 2020, with an overall average of 30.9%.

**Figure 4:**
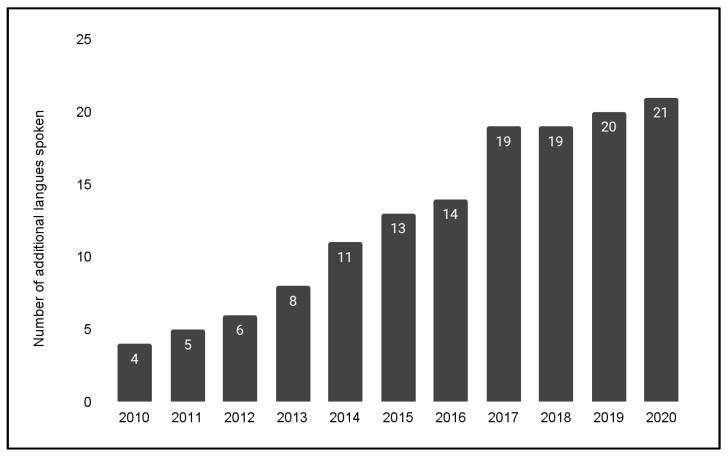
Additional languages (not English or Spanish) spoken by patients and family. The number of additional languages spoken has increased over time from a total of *n* = 4 in 2010 to a total of *n* = 20 in 2020, mainly among Black or Asian patients.

Finally, to determine if the increase in Black and Asian patients and the decrease in Hispanic patients and Spanish speakers were associated with any changes in preferred (non-English, non-Spanish) language spoken, these data were graphed over time (Figure 4). The data demonstrated that the overall number of additional languages spoken (non-English and non-Spanish) has increased over time from a total of *n* = 4 in 2010 to a total of *n* = 21 in 2020—an increase of more than five-fold. In addition, the majority of these additional languages were found among patients that self-identified as Black (African) or Asian.

To determine which additional languages were spoken and if they corresponded with patients of Asian or African descent, each spoken language was sorted by year (Table 2). These data demonstrated that although there were a number of European languages spoken, many of those were languages that required translation, including Albanian (ALB), Armenian (ARM), Dutch (DUT), French (FRE), Latvian (LAT), Portuguese (POR), Russian (RUS), and Slovenian (SLO). Several African languages were also the preferred language for many patients and guardians, including Afar (AFA), Afrikaans (AFR), Amharic (AMH), Arabic (ARA), and Ethiopian (ETH). Finally, a large number of Asian languages were also reported, including Abkhazian (ABK), Chinese (CHI) Mandarin, Dzongkha (DZO), Korean (KOR), Persian (PER), Pushto/Pashto (PUS), Thai (THA), and Tagalog (TAG).

Finally, the data regarding enrollment in Medicaid or the Children’s Health Insurance Program (CHIP) were evaluated (Table 3). These data demonstrated that the percentage of pediatric patients enrolled in Medicaid/CHIP was fairly constant over time, varying between 76.7% in 2010 and 77.9% in 2020, which demonstrates that this patient population is primarily low-income patients enrolled in some form of public assistance program.

## 4. Discussion

The primary objective of this study was to analyze relevant information regarding the pediatric patient population at a public dental school clinic in order to determine any significant changes that might require additional resources or attention. This study successfully determined the demographic characteristics of this population, which are predominantly focused on minority children within the local community [19,20]. Although some recent studies have identified changes in oral microbial prevalence among this patient population [35,36,37], this study identified two recent trends, which suggest additional barriers to access might need to be addressed.

For example, these data revealed that the percentage of Black and Asian patients increased in recent years, which corresponded with a decrease in Hispanic patients. These data do not suggest an overall decrease in the proportion of minority or low-income patients, which are the main risk factors associated with poor oral health and disease among this patient population [38,39,40]. However, these data did also reveal a significant increase in the number of non-English and non-Spanish speaking patients (and guardians) from Asian and African communities. These data did represent a significant shift over time with the number and range of languages spoken and the number of limited English proficiency (LEP) patients needing an interpreter increasing more than five-fold over the period evaluated, which has previously been associated with health disparities and barriers to treatment and care [41,42].

Although some efforts to promote oral healthcare and dental access in LEP communities have been made through the use of leaflets and websites targeting ethnic and regional minorities, improving and enhancing the treatment of linguistically different patients has become the focus of more recent research and public health efforts [43,44]. These studies have revealed the difficulties of treating patients using over-the-phone translation services, as well as problems associated with a lack of appropriate word choice with non-technical language translation services such as Google Translate [45,46]. In fact, native language spoken has more recently been identified as a risk factor for dental disease and tooth decay, suggesting that additional research and resources may be needed to provide culturally and linguistically appropriate care for the patients being served at these clinics [47].

Although many recent community-based coalitions have been formed to address the healthcare disparities among ethnic and racial minorities in the US in recent years, more particular attention may be needed to address the more specific barriers to access and care faced by LEP or linguistically challenged patients and their guardians [48,49,50]. Newer modules and continuing education workshops that focus more specifically on how to work with interpreters in healthcare teams have been demonstrated to improve oral health outcomes and reduce disparities within this patient population [51,52,53]. This study may be the first to identify the growing need for these types of training and workshops, which may be useful to meet the oral health needs of this patient population.

For example, this study found an increasing percentage of patients with limited language proficiency over time (0.26% to 1.44%), which also included an increasing diversity of languages spoken (*n* = 4 to *n* = 21) over the same time period. These data are significant as recent studies have shown that limited language proficiency may be increasingly associated with gaps in healthcare coverage, lower levels of insurance and public assistance qualification, as well as decreased measures of oral health [54,55,56]. In fact, many studies have confirmed that non-English, non-Spanish speaking patients and households experience additional barriers and needs beyond those typically associated with low-income and low-SES populations, which may suggest additional resources are needed to reach them in language- and culturally appropriate ways [57,58,59].

## 5. Conclusions

Although the overall percentage of UNLV-SDM pediatric patients receiving Medicaid or CHIP support has not changed significantly over the past decade, the composition of the patient population has experienced significant shifts with fewer Hispanics and more patients of mixed racial backgrounds. This may be the first report to determine that the number of foreign languages spoken by children and parents has increased dramatically in this dental clinic between 2010 and 2020. These same demographic shifts at UNLV-SDM may suggest that multilingual health materials, translators, and training on how to work with LEP patients and guardians may be needed to design and implement appropriate prevention and treatment strategies for pediatric oral health. Future studies from this group may focus on the need for consistent and accurate data collection about pediatric patients and parents with LEP, as well as the role of referrals for translation services and English classes, which may help to reduce healthcare disparities and barriers to access among this patient population.

## Figures and Tables

**Table 1:** Demographic analysis of study population.

	**Females**	**Males**	**Statistical Analysis**
Complete data: Sex 98.4% (*n* = 24,460/24,849)	52.2% (*n* = 12,758/24,460)	47.9% (*n* = 11,702/24,460)	X^2^ = 0.160 d.f. = 1 *p* = 0.6892
	**White/Caucasian**	**Minority/non-White**	**Statistical analysis**
Complete data: Race 77.6% (*n* = 19,281/24,849)	19.3% (*n* = 3713/19,281)	80.7% (*n* = 15,568/19,281)	X^2^ = 31.473 d.f. = 1 *p* = 0.0001
		Hispanic 52.4% (*n* = 10,110/19,281) Black 12.2% (*n* = 2347/19,281) Asian 3.8% (*n* = 741/19,281) Native American 0.1% (*n* = 26/19,281) Mixed Race 12.2% (*n* = 2344/19,281)	X^2^ = 40.243 d.f. = 4 *p* = 0.0001
	**Mean (average) age**	**Range age**	**Age distribution**
Complete data: Age 81.9% (*n* = 20,361/24,849)	9.04 years +/−4.86 years (STD)	1–18 years	0–2 years 10.1% (*n* = 2052/20,361) 3–4 years 11.1% (*n* = 2268/20,361) 5–6 years 12.6% (*n* = 2557/20,361) 7–8 years 14.4% (*n* = 2941/20,361) 9–10 years 13.4% (*n* = 2719/20,361) 11–12 years 11.4% (*n* = 2328/20,361) 13–14 years 9.8% (*n* = 1987/20,361) 15–16 years 8.9% (*n* = 1832/20,361) 17–18 years 8.2% (*n* = 1677/20,361)

**Table 2:** Preferred languages spoken (not English or Spanish).

Year	European Language	African Language	Asian Language
2010 LEP = 0.26%	**Dutch (DUT)** **Slovenian (SLO)**		**Tagalog (TAG)**
2011 LEP = 0.26%	Dutch (DUT) Slovenian (SLO)	**Ethiopian (ETH)**	Tagalog (TAG)
2012 LEP = 0.31%	Dutch (DUT) Slovenian (SLO)	Ethiopian (ETH)	**Chinese (CHI) man.** Tagalog (TAG)
2013 LEP = 0.36%	**Armenian (ARM)** Dutch (DUT) Slovenian (SLO)	Ethiopian (ETH)	Chinese (CHI) mand. **Korean (KOR)** **Persian (PER)** Tagalog (TAG)
2014 LEP = 0.61%	Armenian (ARM) Dutch (DUT) **Portuguese (POR)** Slovenian (SLO)	**Arabic (ARA)** Ethiopian (ETH)	Chinese (CHI) mand. Korean (KOR) Persian (PER) Tagalog (TAG)
2015 LEP = 0.82%	Armenian (ARM) Dutch (DUT) **French (FRE)** Portuguese (POR) Slovenian (SLO)	Arabic (ARA) Ethiopian (ETH)	Chinese (CHI) mand. Korean (KOR) Persian (PER) Tagalog (TAG)
2016 LEP = 0.91%	**Albanian (ALB)** Armenian (ARM) Dutch (DUT) French (FRE) Portuguese (POR) Slovenian (SLO)	**Afar (AFA)** **Amharic (AMH)** Arabic (ARA) Ethiopian (ETH)	**Abkhazian (ABK)** Chinese (CHI) mand. Korean (KOR) Persian (PER) Tagalog (TAG)
2017 LEP = 1.23%	Albanian (ALB) Armenian (ARM) Dutch (DUT) French (FRE) **Latvian (LAT)** Portuguese (POR) **Russian (RUS)** Slovenian (SLO)	Afar (AFA) **Afrikaans (AFR)** Amharic (AMH) Arabic (ARA) Ethiopian (ETH)	Abkhazian (ABK) Chinese (CHI) mand. Korean (KOR) Persian (PER) **Pushto/Pashto (PUS)** **Thai (THA)** Tagalog (TAG)
2018 LEP = 1.23%	Albanian (ALB) Armenian (ARM) Dutch (DUT) French (FRE) Latvian (LAT) Portuguese (POR) Russian (RUS) Slovenian (SLO)	Afar (AFA) Afrikaans (AFR) Amharic (AMH) Arabic (ARA) Ethiopian (ETH)	Abkhazian (ABK) Chinese (CHI) mand. Korean (KOR) Persian (PER) Pushto/Pashto (PUS) Thai (THA) Tagalog (TAG)
2019 LEP = 1.24%	Albanian (ALB) Armenian (ARM) Dutch (DUT) French (FRE) Latvian (LAT) Portuguese (POR) Russian (RUS) Slovenian (SLO)	Afar (AFA) Afrikaans (AFR) Amharic (AMH) Arabic (ARA) Ethiopian (ETH)	Abkhazian (ABK) Chinese (CHI) mand. **Dzongkha (DZO)** Korean (KOR) Persian (PER) Pushto/Pashto (PUS) Thai (THA) Tagalog (TAG)
2020 LEP = 1.44%	Albanian (ALB) Armenian (ARM) Dutch (DUT) French (FRE) Latvian (LAT) Portuguese (POR) Russian (RUS) Slovenian (SLO)	Afar (AFA) Afrikaans (AFR) Amharic (AMH) Arabic (ARA) Ethiopian (ETH)	Abkhazian (ABK) Chinese (CHI) mand. Dzongkha (DZO) Korean (KOR) Persian (PER) Pushto/Pashto (PUS) Thai (THA) Tagalog (TAG)

Note: New languages are bolded the first time they appear in the clinic charts. Limited English Proficiency (LEP) was calculated based upon the number of patients with specific chart notations for other languages spoken, compared with the total number of patients with any language notation (e.g., English or Spanish). Note: each year also contained patients who are deaf (non-hearing), which was denoted by SIGN (requiring sign language).

**Table 3:** Comparison of Pediatric Medicaid and CHIP enrollment over time.

Year	Percentage (%) of Children on Medicaid/CHIP at UNLV-SDM
2010	76.7%
2011	77.2%
2012	76.9%
2013	77.1%
2014	77.3%
2015	73.1%
2016	76.8%
2017	77.4%
2018	77.6%
2019	77.2%
2020	77.9%

## Data Availability

The data presented in this study are available on request from the corresponding author. The data are not publicly available due to the study protocol data protection parameters requested by the IRB and OPRS for the initial study approval.
